# Profile of asthmatic patients assisted on the emergency of Hospital Geral de Nova Iguaçu (HGNI), NOVA IguaÇu, Brasil

**DOI:** 10.1186/1939-4551-8-S1-A97

**Published:** 2015-04-08

**Authors:** Sérgio Duarte Dortas, Daniella Mouta Dos Santos Silva, Luiz Gustavo Bernardo De Oliveira, Gustavo Henrique De Martin Ballini, Raphael Coelho, Aniele Soares Moritz, Sylvia Gabriella Maia Araújo, Gisele Cardoso Nery

**Affiliations:** 1 Universidade Iguaçu, Brazil

## Background

Asthma is a highly prevalent disease on the emergency services. However, is notable the difficulty of the public services to deal with this type of patients, since part of them have recurrent exacerbations.

We aim to describe the profile of the patients older than 12 years old admitted to a regional reference emergency department (ED) presenting bronchospasm (ICD: J45 and J42) in the HGNI from January/2008 to March/2014.

## Methods

A descriptive cross-sectional study with retrospective data collection from patients admitted in the emergency service of HGNI presenting bronchospasm between 2008 and 2014. Parameters analyzed were: gender, age, race, period of hospitalization, severity of the exacerbation, gestation, complications, medications used on the treatment and readmissions.

## Results

Data from 35 patients older than 12 years old (mean age - 40 years old) were collected. There was a predominance of patients aged 20-40 years. Prevalence was higher in females (83%), 45% of them were pregnant woman. As for the race, there was a predominance of brown (63%), followed by white (20%) and black (14%). 80% of patients were hospitalized for less than 10 days. Regarding the severity of exacerbation, the prevalence of mild to moderate attacks (66%), followed by severe crises (20%) and respiratory arrest imminent (14%). The main complication associated was pneumonia (43% of cases). The proposed treatment for all patients was the combination of hydrocortisone and nebulized bronchodilators. The use of aminophylline was reported in 37% of cases. Among all patients, 5 were readmitted (14%) and 4 evolved to death (11%).

## Conclusions

Through our study we noticed the impact of asthma in the emergency department of our hospital, however, we consider that the number of assisted asthmatic patients during these six years was underreported. The results showed the predominance of exacerbations in female adult patients, as reported in previous studies. It is alarming the number of readmissions and deaths during this period, reflecting the lack of maintenance treatment for these patients. Therefore, there is a need to establish a prevention program to educate asthmatic patients and alert to doctors that asthma treatment should be individualized.

